# PARP Inhibitor Resistance Mechanisms and Implications for Post-Progression Combination Therapies

**DOI:** 10.3390/cancers12082054

**Published:** 2020-07-25

**Authors:** Elizabeth K. Lee, Ursula A. Matulonis

**Affiliations:** 1 Department of Medical Oncology, Dana-Farber Cancer Institute, Boston, MA 02215-5450, USA; eklee@partners.org; 2 Division of Gynecologic Oncology, Dana-Farber Cancer Institute, Boston, MA 02215-5450, USA

**Keywords:** ovarian cancer, PARP inhibitor, PARP inhibitor resistance, DNA damage repair, homologous recombination, BRCA, replication fork

## Abstract

The use of PARP inhibitors (PARPi) is growing widely as FDA approvals have shifted its use from the recurrence setting to the frontline setting. In parallel, the population developing PARPi resistance is increasing. Here we review the role of PARP, DNA damage repair, and synthetic lethality. We discuss mechanisms of resistance to PARP inhibition and how this informs on novel combinations to re-sensitize cancer cells to PARPi.

## 1. Introduction

Poly(ADP-ribose)polymerase (PARP) inhibitors (PARPi) have changed the treatment landscape of epithelial ovarian cancers (EOC). PARPi are FDA approved across all lines of treatment of EOC. While this expands the treatment armamentarium, this also raises the question of how best to treat patients who progress on a PARPi. Effectively, these patients are considered PARPi resistant, and there arises an urgent need to understand and clinically validate the mechanisms of PARPi resistance, allowing us to determine appropriately matched post-PARPi-progression combination therapies. Additionally, these strategies will also likely be applicable to homologous recombination proficient (HRP) and BRCA wild-type (BRCAwt) cancers, where only minimal benefit is seen with single agent PARPi. Here, we review the role of PARP in DNA repair pathways, the effects of PARPi, mechanisms of resistance, and strategies for subsequent combination therapies.

## 2. DNA Damage Repair and the Role of PARP

High fidelity DNA repair is integral to cell survival. Therefore, there are several pathways by which DNA damage can be repaired. PARP plays multiple roles in several DNA repair pathways, highlighting where PARPi exerts its cytotoxic function, but also demonstrates the points at which PARPi resistance may arise. 

Double strand breaks (DSBs) in DNA are highly toxic lesions for which there are several DNA repair processes, the choice of which is influenced by the cell cycle phase during which the DSB occurs, the availability of a DNA template, and competition between functional effector proteins [1].

Homologous recombination (HR) is one means of repairing DSBs and is restricted to the S and G2 phases when a sister chromatid is present as a repair template. DSBs are recognized by PARP1, whereupon poly(ADP-ribos)ylation (PARylation) initiates a cascade of protein recruitment, including that of BRCA1 and the MRN complex, composed of the endonucleases MRE11, RAD50, and NBS1. The 3′ strand end-resection is initiated by the MRN complex and is continued by other nucleases, such as CTIP, DNA2, and EXO-1. The resultant overhanging single stranded DNA (ssDNA) is coated by replication protein A (RPA). BRCA1, PALB2, and BRCA2 facilitate the replacement of RPA with RAD51, generating a RAD51-loaded nucleoprotein filament; this subsequently facilitates strand invasion of the sister chromatid and error-free, fidelitous DNA synthesis [2]. 

End-resection is a crucial step in HR and is balanced by antagonism between BRCA1 and 53BP1 [2]. The 53BP1 interacts with the shieldin complex (which includes a REV7 subunit [3,4]) to bind the initial 3′ ssDNA overhangs. The shieldin complex protects against further end-resection, preventing the completion of HR and diverting DNA repair toward the classical non-homologous end-joining (cNHEJ) pathway [3,4,5,6]. Furthermore, DYNL11 (dynein light chain 1) promotes the formation of 53BP1 complexes at DSB sites and represses the activity of the MRN complex [1]. Another negative regulator of HR is DNA helicase B (HELB), which is recruited by RPA to coated ssDNA and represses further end resection, serving as another means to divert DNA repair from HR toward NHEJ.

cNHEJ, the other primary means of DSB repair, is a mechanism that is preferentially employed during the G1 phase [1,7], occurs without a repair template, and is inherently error-prone. Here, the free ends of DNA are bound by the Ku70/Ku80 complex, leading to the recruitment of DNA-dependent protein kinase catalytic subunit (DNA-PKcs) and the formation of a DNA-PK complex that recruits downstream ligation proteins. Competition between PARP1 and the Ku70/80 binding of the free DNA ends underlies the anti-cNHEJ role of PARP [8]. The limited amount of end-processing required for end to end ligation leads to deletions and inaccurate gap-filling and explains the mutagenic nature of NHEJ. 

If the DSB occurs in a region of DNA with areas of microhomology, and if the Ku complex is absent or out-competed for binding, alternative NHEJ (altNHEJ) can be employed for repair. This process involves PARP1-mediated recruitment of the MRN complex [9], alignment of ssDNA at areas of microhomology, and POLθ- and LIG3-mediated gap-filling and ligation. POLθ is an error-prone polymerase that antagonizes RAD51-mediated recombination, thereby suppressing HR [10]. Similarly, DSBs occurring in areas with longer stretches of homology may rely on the single strand annealing (SSA) pathway of repair [11], which is also inherently mutagenic. 

Furthermore, PARP1 functions to repair single strand breaks (SSBs), is involved in base excision repair (BER) [12,13] and nucleotide excision repair (NER) [9,14], and promotes nucleosome dissociation to allow chromatin relaxation [15,16].

## 3. Replication Fork Dynamics and the Role of PARP

PARP1 has been shown to modulate replication fork dynamics in situations of replication stress. PARP1 binds the sites of DNA damage, forming a PARP–DNA complex. Replication forks that encounter these complexes become stalled and undergo fork reversal. In this process, nascent ssDNA overhangs are protected by RAD51 filaments, thought to be facilitated by BRCA1 and BRCA2 [2,17]. PARylated PARP, bound to damaged DNA, concomitantly inhibits the DNA helicase Q1 (RECQ1) to prevent premature fork restart [9]. Once the DNA lesion is repaired and PARP1 has dissociated, RECQ1 is no longer inhibited and promotes replication fork restart. There is conflicting evidence that limited end-resection may be required for fork restart, partly facilitated by PARP1-mediated recruitment of MRE11 [9,18]. If the coordination of these steps does not occur, progression of the replication fork into DNA lesions causes fork collapse and the generation of toxic DSBs.

## 4. Effects of PARP Inhibitors 

PARPi can be highly injurious to a cancer cell due to its multiple roles in DNA repair and synthesis, which is tightly coordinated with the cell cycle. The deleterious effects of PARPi are further amplified in the context of underlying alterations affecting DNA repair or cell cycle regulation, such as in high grade serous ovarian cancer (HGSOC), the most common EOC histologic subtype. Approximately 50% of HGSOC are HR deficient [19,20], comprising germline (14%) and somatic (6%) BRCA1/2 mutations, BRCA1 promoter methylation (10%) and RAD51C promoter methylation and other *RAD* mutations (3–4%). Additionally, upwards of 90% of HGSOC patients harbor mutant *TP53*, which is critical for the appropriate regulation of the cell cycle, acting primarily at the G1/S checkpoint but also the G2/M checkpoint [21,22,23,24,25]. HGSOC is, therefore, particularly susceptible to additional perturbations in the DNA repair process, such as by PARPi.

This forms the basis for synthetic lethality with PARPi [19,26,27]. For example, PARP inhibition allows the accumulation of unrepaired SSBs, which are processed into DSBs during replication. In the absence of a functional HR pathway, and in the setting of unopposed Ku70/80 binding, DSBs are shunted toward error-prone cNHEJ. The accumulation of DNA errors leads to progressive genomic instability and cell death. Replication fork dynamics are also altered. PARPi cause the trapping of PARP1 at sites of DNA lesions, wherein the dissociation of PARP1 is impaired [28]. Without the resolution of these lesions, replication forks remain stalled. PARPi also disrupt the careful coordination of MRE11 and RECQ1, allowing strand degradation and inappropriate fork restart, leading to fork collapse. Furthermore, unresolved DNA lesions carried through a dysregulated cell cycle can precipitate mitotic catastrophe and cell death.

## 5. Biomarkers of PARP Inhibition

Determining predictive biomarkers of response to PARP inhibition is an area of significant interest and continued investigation [29,30]. The presence of a BRCA mutation [31] or a “BRCA-like” gene expression profile [32] both correlate with PARPi response. Among patients with a germline BRCA mutation, platinum sensitivity and fewer prior lines of therapy were associated with higher response rates and longer durations of response to PARPi [33]. The measurement of HR deficiency, based on genomic characteristics, such as loss of heterozygosity or telomeric allelic imbalance, are utilized in commercial assays, such as the Myriad myChoice HRD assay, and also appear to correlate with PARPi response [29]. Whether level of PARP1 expression correlates with response to PARPi remains under investigation. One study evaluating primary ovarian cancer samples found no correlation [34], whereas a radiotracer-PARP1 study found a significant correlation with response to two PARPi [35]. 

## 6. PARP Inhibitors in Ovarian Cancer

The clinical use of PARPi in the treatment of EOC has expanded dramatically. Olaparib, rucaparib, and niraparib were initially approved for use in the recurrence setting as monotherapy [36,37,38] agnostic of sensitivity to platinum, followed by approval as post-chemotherapy maintenance for platinum sensitive disease [39]. PARPi are now FDA approved as frontline maintenance. Olaparib obtained FDA approval in 2018 as maintenance following response to frontline platinum-based therapy for patients with germline or somatic BRCA-mutated EOC [40]. In April 2020, niraparib received FDA approval as maintenance following response to frontline platinum regardless of HR status [41], and the combination of olaparib/bevacizumab received FDA approval in May 2020 as maintenance for patients with HRD EOC [42]. 

Analysis of the frontline PARPi maintenance studies may yield the best indicators of baseline rates and kinetics of de novo and acquired PARPi resistance, as this population is the least affected by prior lines of treatment. The randomized placebo-controlled phase III SOLO-1 trial studied maintenance olaparib following partial or complete responses to platinum-based frontline chemotherapy in patients with germline or somatic BRCA-mutated advanced HGSOC or high grade endometrioid EOC [43]. Olaparib maintenance was estimated to improve median PFS by approximately 36 months, as median PFS had not yet been reached at the time of data reporting. However, the continued negative slope of the Kaplan–Meier survival curve for patients receiving olaparib highlights disease recurrence despite PARPi treatment. Similarly, the randomized placebo-controlled phase III PRIMA/ENGOT-Ov26 trial studied niraparib in the frontline maintenance setting in patients with or without a known BRCA mutation or evidence of HRD by the Myriad myChoice assay [44]. Median PFS improved from 8.2 months to 13.8 months with niraparib maintenance in the overall population, and prespecified molecular subgroup analysis demonstrated that the benefit was greatest in those with BRCA mutations (median PFS 22.1 versus 10.9 months), followed by those with non-BRCA HR deficiency (19.6 versus 8.2 months). Regardless of this susceptibility, however, disease recurrence substantiates the development of PARPi resistance.

## 7. Mechanisms of PARP Inhibitor Resistance 

**Figure 1:**
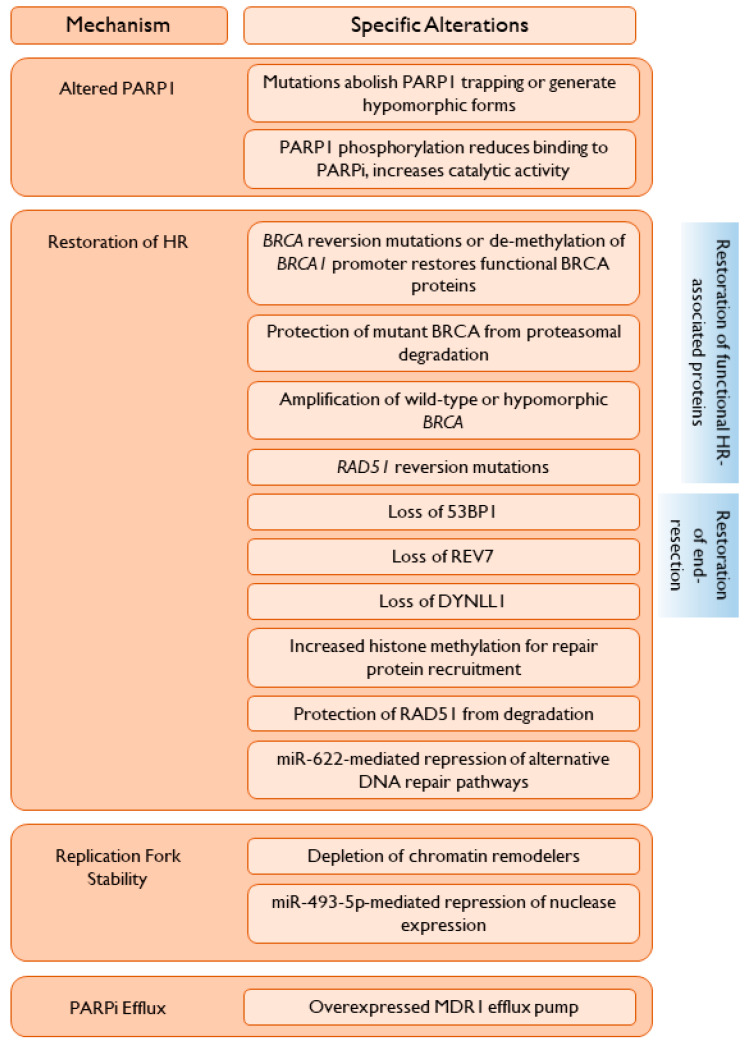
Thematic mechanisms of resistance to PARP inhibition.

The increase in PARPi use will be paralleled by an increasing number of women who are found to have de novo or acquired resistance to PARPi. As seen in many trials evaluating PARPi as post-chemotherapy maintenance or as monotherapy, a substantial proportion of women progress on treatment, highlighting an urgent need to determine appropriate post-progression treatment, the choice of which may be influenced by the mechanism(s) of PARPi resistance [45]. Select resistance mechanisms (summarized in Figure 1) are discussed in detail below.

### 7.1. Alterations in PARP1

#### 7.1.1. PARP1 Mutations

Mutations in PARP1 can diminish the binding of PARPi or allow PARP1 to maintain endogenous functions. A large-scale Crispr-cas9 mutagenesis screen with in vitro clonal selection following PARPi selection pressure identified in-frame mutations occurring within the DNA-binding zinc-finger domain of PARP1 [46]. Mutations were frequently seen involving residues K119 and S120 and the surrounding region, impacting the ability of PARP1 to bind sites of DNA damage. This suggests that the abolishment of PARP1 trapping underlies those clones’ resistance to PARPi. A mutation arising in a region predicted to contribute to the DNA-binding interface abolished PARP1 trapping, as did a separate unique mutation located in the PARP1 regulatory region. A catalytic domain mutation yielded a mutant PARP1 that had retained but diminished recruitment to sites of DNA damage, PARylation, and DNA binding capacity. In response to PARPi, there was only transient PARP trapping. These preclinical data highlight that PARP1 mutations altering PARP trapping may serve as a mechanism of PARPi resistance. This hypothesis is supported by a reported case of a patient with EOC and de novo resistance to olaparib, who was subsequently found to have a PARP1 mutation affecting a region critical for the communication between the DNA-binding and catalytic domains [46]. The resulting PARP1 protein retained DNA-binding capacity but was unable to become trapped in response to PARPi. 

#### 7.1.2. Post-Translational Modifications of PARP1

Post-translational modifications of PARP1 may also impact its function and confer PARPi resistance. Preclinical studies in models of triple negative breast cancer found that MET, activated in response to oxidative DNA damage, was able to phosphorylate PARP1 [47]. This modification resulted in reduced binding by PARPi, an increase in PARP1 enzymatic activity, and resistance to PARPi. Cells were re-sensitized to PARPi with the use of a MET inhibitor, and dual MET and PARP inhibition led to synergistic anti-tumor activity in breast and lung cancer xenografts. In contrast, in a separate model of hepatocellular carcinoma, PARP1 phosphorylation was dependent on the nuclear translocation of EGFR and formation of an EGFR/MET heterodimer causing MET activation [48]. Whether PARP1 phosphorylation in EOC relies on MET alone or requires another receptor tyrosine kinase, such as EGFR, is unknown.

### 7.2. Restoration of Homologous Recombination 

A common mechanism of resistance to PARPi is the restoration of at least partial HR capabilities, such that DSBs can be repaired, decreasing genomic instability and replication stress. This can occur through the restoration of a deficient HR-related protein to a functional capacity, or by the alteration of inhibitory proteins such that HR can proceed (Figure 1).

#### 7.2.1. Restoration of Functional HR-Associated Proteins

##### Reversion Mutations

Somatic BRCA reversion mutations are a well described mechanism of resistance to PARPi, occurring in approximately 20% of cases of EOC [49,50]. As pathogenic BRCA1/2 mutations may lead to frameshifts, protein truncation, and the generation of a hypomorphic or non-functional protein, reversion mutations can be insertions or deletions that restore the open reading frame, remove the original deleterious mutation to restore a nearly full-length wild-type sequence, or cause a synonymous mutation, restoring wild-type amino acid sequences [51,52]. This results in the restoration of a functional BRCA protein [50,51,53,54,55,56,57,58,59]. Micro-homology associated with reversion alterations suggest that they arise as a result of the error-prone altNHEJ or SSA pathways of DNA repair, utilized in the setting of HR deficiency [53,54,60]. Multiple concurrent reversion mutations may evolve following PARPi treatment, representing multiple subclones [49,54,56,59,61,62] that may contribute to clinically heterogenous intra-patient progression of disease. In one case, 12 separate reversion mutations were found within one patient who developed resistance to rucaparib [61].

Reversion mutations are similarly found in other altered HR-associated proteins. For example, reversion mutations in RAD51C and RAD51D have been described in association with acquired resistance to rucaparib in a cohort of HGSOC patients from the ARIEL2 trial [63,64].

##### BRCA1 Promoter Alterations

The de-methylation of BRCA1 leading to the re-expression of protein and restored HR was demonstrated in patient-derived xenograft (PDX) models of PARPi-resistant breast cancer [60]. This was reported clinically, with BRCA1 de-methylation occurring at the time of relapse in a patient with PARPi resistant EOC [56] and in two patients following neoadjuvant platinum-based chemotherapy [65]. The zygosity of BRCA1 methylation appears to be important for PARPi sensitivity or resistance, with heterozygous methylation correlating with PARPi resistance in HGSOC PDX models [65]. Additionally, BRCA1 promoter methylation can be subverted by intrachromosomal rearrangements that place BRCA1 expression under the control of a different promoter, as was found in PARPi-resistant PDX tumors [60].

##### Generation of Hypomorphic BRCA Proteins

Alternative splicing of BRCA1 can generate a hypomorphic protein with residual function. The expression of the BRCA1Δ11q splice variant, with partial skipping and exclusion of most of the exon 11 nucleotides, was associated with in vitro and in vivo PARPi resistance [66]. Mutations in the highly conserved RING domain of BRCA1, crucial for interaction with BARD1, yielded a protein that demonstrated increased stability independent of BARD1, with retained function and PARPi resistance [67]. Furthermore, RING domain deficient BRCA1 was detected in patient derived specimens with germline BRCA1 mutations, confirming this as a clinically relevant mechanism of resistance. Similarly, the generation of hypomorphic BRCA1 can result from mutations occurring in close proximity to Alu elements in intron 15 of BRCA1 [68]. These mutant BRCA1 isoforms were able to avoid proteasomal degradation, retained partial RAD51-loading capability despite loss of the BRCT domain, and promoted PARPi resistance [68]. Additionally, the authors determined that BRCA1 gene rearrangements led to the translation of intron 15. This BRCA1 isoform was functional, retained RAD51 loading ability, and also generated PARPi resistance in vitro [68].

##### Decreased Proteasomal Degradation

Mutations within the BRCT domain itself can create a misfolded protein, which typically undergoes proteasomal degradation [69,70]. However, under PARPi selection pressure in vitro, PARPi resistant cells were found to have increased expression of the mutant BRCA1 due to Hsp90-mediated stabilization [71]. These BRCT-mutated proteins were functional and able to interact with PALB2-BRCA2 for RAD51 foci formation, thereby engendering PARPi resistance. Analysis of specimens obtained from platinum-treated recurrent BRCA1-mutated EOC patients found that, of four patients with BRCT domain mutations, two patients had increased BRCA1 protein expression in the absence of reversion mutations. Analogous to the in vitro data, this was likely due to Hsp90-mediated stabilization [71].

##### Amplification of Wild-Type BRCA

Copy number gain or upregulation of the wild-type, functional BRCA allele was found to underlie the PARPi resistance in a cohort of patients with HGSOC [72]. In one patient, the primary tumor had single-copy loss of the majority of chromosome 17q. However, at the time of progression, there was single-copy gain of the remaining wild-type allele, resulting in two copies of the non-mutated BRCA1 allele. A second patient developed upregulation of the wild-type BRCA2 allele at time of progression. However, in vitro analysis of BRCA2-mutant PARPi resistant clones found that amplification and copy number gain of even the mutant BRCA2 allele was sufficient for the restoration of HR and PARPi resistance. Therefore, this may be a mechanism of resistance in situations of a hypomorphic BRCA mutation [73]. 

#### 7.2.2. Restoration of End-Resection

A crucial step in HR is end-resection, initiated by the MRN complex and extended by additional nucleases, including EXO-1 and DNA2. Alterations in proteins associated with end-resection may restore HR capability in previously HR-deficient cancers, leading to PARPi resistance (Figure 1).

Loss of 53BP1, a repressor of end-resection, was shown in vitro to partially restore HR despite BRCA1 deficiency, conferring PARPi resistance [74,75]. The loss of 53BP1 was seen in 20% of PARPi-resistant breast cancer PDXs [76]. One patient with PARPi-resistant BRCA-mutant breast cancer, identified through genomic analysis of a breast cancer cohort, was found to have biallelic inactivation of TP53BP1 [77]. How the loss of 53BP1 restores HR is likely manifold. The loss of 53BP1-mediated suppression of ATM-dependent RPA phosphorylation rescues RPA loading onto ssDNA. Concomitantly, 53BP1 loss appears to abrogate ATM-mediated cell cycle checkpoint arrest, allowing the restoration of cell proliferation [78,79]. Lastly, if BRCA1 is deficient and cannot facilitate PALB2 recruitment, the loss of 53BP1 allows the exposure of the nucleosome region required for PALB2 self-localization and direct DNA binding [75].

The restoration of HR due to the loss of 53BP1 appears specific to BRCA1 but not BRCA2 deficiency [78], with the type of BRCA1 mutation dictating the extent to which HR is restored. For example, in cells with 53BP1 loss and BRCA1 mutations not affecting the coiled-coil domain (required for interacting with PALB2), HR was restored to a greater degree compared to BRCA1 with disrupted coiled-coil domains [80,81,82]. Similarly, the depletion of REV7, a component of the shieldin complex, was found in vitro to restore HR through CtIP-mediated end-resection, leading to PARPi resistance. This was seen in BRCA1-, but not BRCA2-, deficient cells, highlighting the different steps in HR in which each BRCA protein acts [83]. 

DYNLL1 suppresses the activity of several components of the end-resection machinery involved in HR, including the MRN complex, thereby limiting end resection. The loss of DYNLL1 permitted the uninhibited recruitment of end-resection proteins to areas of DNA damage, allowing end resection and HR to proceed [84]. Even in BRCA1 mutant cells, DYNLL1 loss enhanced end-resection, restored RAD51 foci formation, and restored HR-mediated DSB repair. This was correlated clinically in a cohort of HGSOC specimens in which BRCA1-deficient cancers with low expression of DYNLL1 were associated with fewer chromosomal abnormalities [84]. In vitro, concurrent DYNLL1 loss and BRCA1 deficiency resulted in resistance to PARPi. In effect, the loss of DYNLL1 compensated for BRCA1 deficiency. 

#### 7.2.3. Promotion of Repair Protein Recruitment

Preclinical data suggest that PARPi resistance may arise from adaptive epigenetic changes. For example, the overexpression of the histone methyltransferases EHMT1/2 was found in PARPi resistant HGSOC [85]. EHMT1/2 promote the recruitment of DNA damage repair proteins [86,87], including those related to HR, and have been implicated in replication fork stability [88], suggesting two downstream mechanisms of resistance. The inhibition of EHMT1/2 ablated markers of HR and NHEJ in PARPi resistant but not sensitive cells, suggests that PARPi resistant cells developed a reliance on EHMT1/2-facilitated repair [85]. In both BRCA-deficient and -proficient cells, the inhibition of EHMT1/2 re-sensitized previously resistant cells to olaparib [85].

In PARPi resistant cells, RAD51 levels are elevated due to the downregulation of EMI1, a mitotic regulator that assembles a ubiquitin ligase complex involved in RAD51 degradation [89]. Therefore, EMI1 downregulation allows the accumulation of RAD51, which may facilitate HR and PARPi resistance [90].

#### 7.2.4. Repression of Alternative DNA Repair Pathways 

If alternative DNA repair pathways are suppressed, HR may be preferentially employed instead. This was demonstrated with microRNA-622 (miR-622), which clinically is associated with worse overall and disease-free survival in BRCA1 mutated cases of EOC [91]. This association was not seen in cases with wild-type BRCA. Mechanistically, miR-622 targeted and decreased the expression of Ku80, suppressing NHEJ. In parallel, this allowed the unfettered recruitment of the MRN complex to DSBs and rescue of HR in BRCA1 mutant cancers, leading to PARPi resistance [91]. The upregulation of Wnt/β-catenin signaling, which may occur secondary to the methylation of FZD10, a receptor in the Wnt pathway [92], also induced PARPi resistance in vitro [93]. Functional assays demonstrated that HR was primarily enhanced, however NHEJ was also promoted. The inhibition of β-catenin significantly impeded HR and NHEJ and restored sensitivity of cells to PARPi [92,94]. This may occur through the modulation of LIG4 by β-catenin, which is involved in NHEJ and upregulation of MRE11 [95,96].

### 7.3. Alteration of Replication Fork Dynamics 

One means of PARPi cytotoxicity occurs through the dysregulation of replication fork reversal and/or restart. Therefore, resistance to PARPi can arise through the stabilization of replication forks [97].

MRE11 is recruited to stalled replication forks and may be involved in limited resection required for fork restart. However, in the setting of BRCA1/2 deficiency it may also precipitate uncontrolled degradation and fork collapse. Fork remodeling is required for MRE11-dependent nascent DNA degradation. This process is facilitated by SMARCAL1, ZRANB3, and HLTF; depletion of these chromatin remodelers prevented strand degradation by MRE11, leading to fork stability, reduced replication stress-induced DNA damage and chromosomal instability, and resistance to olaparib in BRCA1/2 deficient cells [98]. Additionally, the loss of the recruitment protein PTIP protects nascent DNA strands from extensive degradation by MRE11, as can occur when BRCA1/2 is deficient or nonfunctional and cannot recruit protective RAD51 [97]. Comparably, MRE11 inhibition reduced the levels of PARPi-induced chromosomal abnormalities, likely through replication fork protection. 

E2F7, a transcription factor induced by DNA damage and involved in facilitating G1/S arrest, also impacts MRE11 activity [99]. E2F7 represses the expression of key HR contributors, including RAD51 and BRCA1 [100]. In vitro depletion of E2F7 led to increased RAD51 levels, restored RAD51-mediated HR repair, and increased stability of stalled replication forks by preventing MRE11-mediated degradation, overall imparting resistance to PARPi.

Similarly, the recruitment of the nuclease MUS81 by EZH2-directed histone methylation facilitates fork restart in BRCA2-deficient cells [101]. Low EZH2 levels reduced MUS81 recruitment and led to fork stabilization. EZH2 inhibition and MUS81 loss each conferred PARPi resistance in BRCA2-deficient cells and breast cancer model.

The nuclear protein SLFN11 detects replication stress, stalls fork progression, and prolongs S phase arrest to allow the repair of DNA lesions prior to the continuation of the cell cycle. However, prolonged fork stalling eventuates replisome disassembly and fork breakage, contrarily predisposing cells further to the genome-destabilizing effects of PARPi. High SLFN11 expression was associated with increased sensitivity to PARPi [102]. Loss of SLFN11 was associated with resistance to PARPi in BRCA1/2-deficient cells [102,103]. 

In keeping with the evidence above, miR-493-5p induced PARPi resistance by decreasing protein expression of the end-resection proteins EXO1, MRE11, BLM, and CHD4 in BRCA2-mutant cells [104]. These factors, recruited by PTIP, are involved in destabilizing the replication fork with additional roles in HR and single strand annealing [17,18,97,105]. Therefore, miR-493-5p promotes fork stabilization and diminishes the repair of DSBs via the mutagenic SSA pathway. In BRCA2-mutated EOC cell lines, miR-493-5p upregulation was associated with olaparib resistance [104].

### 7.4. PARPi Efflux 

Upregulation of drug efflux pumps is a well described mechanism of PARPi resistance. Gene alterations in ABCB1, encoding the multi-drug efflux pump MDR1 (also known as p-glycoprotein), include intergenic deletions, transcript fusions, and 5′ region mutations, leading to the increased expression of ABCB1 [56,106]. Transcript fusions can place ABCB1 under the control of heterogeneous genes and promoters. In one study of patients with recurrent HGSOC, fusions accounted for 59% of the specimens with the highest MDR1 expression [106]. Approximately 8% of HGSOC specimens, taken at time of post-PARPi recurrence, found the upregulation of ABCB1 via fusions and translocations [56].

Of note, paclitaxel and doxorubicin are also MDR1 substrates. Theoretically, the use of either agent prior to PARPi may similarly induce MDR1 upregulation and indirectly induce PARPi resistance. In one study, prior paclitaxel use was significantly associated with the presence of ABCB1 fusion transcripts [106]. However, prior doxorubicin use was not significantly associated with ABCB1 fusion transcripts. The association of MDR1 cross-resistance between PARPi and paclitaxel has been demonstrated in additional studies [107,108]. Therefore, conversely, MDR1 overexpression occurring as a PARPi resistance mechanism has implications for which agent to use following PARPi resistance.

## 8. Implications for Post-Progression PARP Inhibitor Combination Therapies 

**Figure 2:**
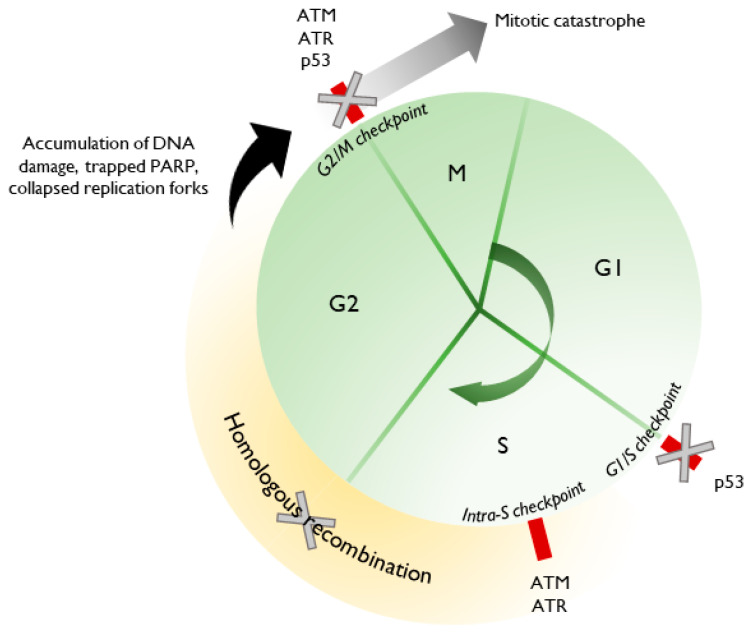
Rationale for re-sensitization to PARPi. Almost all HGSOC harbor mutant p53, with cell cycle dysregulation at baseline. Inhibition of ATM or ATR further prevents the appropriate halting of the cell cycle. Using agents that reestablish HR deficiency allows for the accumulation of endogenous and exogenous DNA damage, trapped PARP, and replication stress, ultimately leading to mitotic catastrophe and cell death.

Barring alterations in PARP1 or increased efflux, PARPi resistance generally occurs via the restoration of HR or replication fork stabilization. Therefore, combination therapies may aim to target one or both to re-sensitize resistant cells to PARPi or to induce PARPi sensitivity in EOC that is HRP at baseline. The rationale behind many combination therapies is depicted in Figure 2. However, many combinatorial strategies are thus far only in the preclinical or early-phase trial stages, where issues, such as additive toxicities precluding therapeutic dosing of one or both agents and lack of comparator arms, hinder the ability to robustly evaluate these combinations [109].

### 8.1. PARPi and Anti-Angiogenic Agents

Anti-angiogenic agents have been shown to inhibit BRCA1/2 expression, which may be beneficial in cases of reversion alterations leading to functional proteins. The inhibition of VEGFR3 in vitro decreased levels of BRCA1 and BRCA2 and inhibited cell growth [110]. In the setting of a BRCA2-mutated clone that developed a resistance mutation and expressed a functionally wild-type protein, VEGFR3 inhibition was sufficient to restore chemosensitivity [110]. The inhibition of VEGF and VEGFR2 in organoid models prevented Akt-mediated DNA repair, thereby preventing HR and leading to aberrant NHEJ [111]. Anti-angiogenics are also hypothesized to induce or exacerbate intratumoral hypoxia, which itself is associated with impaired HR [112,113,114,115]. Therefore, preclinical data suggest that combining anti-angiogenic agents with PARPi may be effective in PARPi resistant disease. A single arm phase II trial treated 34 patients with PARPi-resistant EOC with olaparib and cediranib (oral anti-angiogenic), yielding 4 patients who achieved partial responses and 18 patients with stable disease [116]. That this combination was effective even in PARPi-resistant cases may be due to cediranib-mediated suppression of BRCA1/2 and RAD51 expression, both indirectly through induction of hypoxia, and directly through transcriptional repression [117]. In a similar patient population of germline BRCAwt platinum sensitive recurrent EOC, combination cediranib/olaparib had greater activity compared to olaparib alone in post-hoc analyses of a phase II trial, prolonging median PFS from 5.7 months to 23.7 months (*p* = 0.002) and median OS from 23.0 months to 37.8 months (*p* = 0.047) [118]. In prespecified subset analysis in a subsequent phase III trial (GY-004), however, the population with germline BRCAwt platinum sensitive recurrent EOC performed comparably to platinum-based chemotherapy (ORR 64% cediranib/olaparib versus 72% chemotherapy; HR 0.97, 95% CI 0.73–1.30) [119]. Cediranib/olaparib performed better than chemotherapy in those with BRCA-mutated disease (ORR 89% versus 71%, HR 0.55, 95% CI 0.32–0.94). Due to the trial’s hierarchical testing design, cediranib/olaparib did not meet statistical criteria for comparison to olaparib monotherapy. Importantly, the phase II and III trials of cediranib and olaparib did not report the somatic BRCA status of patients. Additionally, the benefit of anti-angiogenic agents may also be influenced by the immunomodulatory effects of anti-VEGF/VEGFR agents [120,121]. 

### 8.2. PARPi and Hsp90 Inhibition

Pairing PARPi with inhibitors of Hsp90 (Hsp90i) may be relevant for EOC with BRCA1 mutations affecting the BRCT domain, given the role of Hsp90 in subverting ubiquitin-directed proteasomal degradation and restoring BRCA1 protein function [71]. Hsp90 stabilization of mutant BRCA1 likely extends beyond BRCT domain mutations [122]. In addition to BRCA1, Hsp90 interacts with several other client proteins involved in DNA repair and cell cycle regulation, including CHK1, BRCA2, RAD51, and MRE11 [122,123]. Therefore, Hsp90i may impair HR by several mechanisms and may additionally impair NHEJ [122]. This was corroborated in preclinical studies using the Hsp90 inhibitor ganetespib. Treatment with ganetespib led to reduced expression of BRCA1, BRCA2, CHK1, ATM, RAD51, MRE11, and CDK1 and was associated with abrogated HR [124]. Pairing ganetespib with talazoparib produced anti-tumor synergy even in BRCAwt, HR-proficient HGSOC cells [124]. This suggests that PARPi resistant cancers with restored HR may be susceptible to combination Hsp90i and PARPi. 

### 8.3. PARPi and PI3K Pathway Inhibition

Impairing DNA damage repair through PI3K inhibition (PI3Ki) occurs through the suppression of BRCA1/2 transcription and depletion of the nucleotide pool [125,126,127,128]. A phase I trial of buparlisib (PI3Ki) with olaparib yielded 12 of 46 patients with EOC who achieved a partial response, of which four patients did not have germline or somatic BRCA mutations [129]. Similarly, a phase Ib trial of alpelisib (PI3Ki) with olaparib in patients with EOC found that 35% (*n* = 6/17) of patients with germline BRCAwt disease achieved partial responses, similar to those with germline BRCA mutations (30%, *n* = 3/10) [130]. Both cohorts were enriched for HR proficient disease, as 94% and 90% of each group, respectively had platinum resistant or refractory disease. In prespecified analyses, archival tumor specimens were assessed for somatic mutations, and in a combined group of germline and somatic BRCAwt disease, 33% of patients (4/12) achieved a response. These data suggest that PI3Ki was sufficient to induce HR deficiency in cancers with baseline HR proficiency and without evidence of PI3K pathway mutations, thereby sensitizing to the effects of PARPi. Though both phase I trials allowed prior PARPi use for patients in dose escalation, response attributions and whether these patients had PARPi resistance is unclear. 

In a phase I trial of olaparib and the AKT inhibitor capivasertib, 11 of 25 patients with EOC achieved clinical benefit (CR + PR + SD ≥ 4 months) [131]. Of these 11 patients, four were PARPi resistant. Despite this, one patient achieved a PR, and two patients achieved prolonged SD of 56 and 115 weeks, respectively. These data suggest that combination therapy was able to re-induce sensitivity to PARPi. Of additional interest is correlative cfDNA analysis that showed BRCA1/2 reversion mutations developing at the time of progression, demonstrating that PARPi resistance can develop despite combination therapy [131]. A separate analysis of patients with endometrial, ovarian, and triple-negative breast cancers treated with olaparib/capivasertib determined that markers of DNA damage checkpoint activation (high phospho-Chk1, -Wee1, -CDC2) and decreased mTOR activity were associated with response, whereas resistance to the combination was associated with high receptor tyrosine kinase activity levels and mTOR activation [132]. Though it is unclear whether markers differed between malignancies or whether any patients included in the analysis had received prior PARPi, these data provide some indication of a molecularly-defined population who may be more likely to respond, and provide greater insight to additional resistance mechanisms.

### 8.4. PARPi and MEK Inhibition

RAS-mutated cell lines were found to be HRP and PARPi resistant, and acquired PARPi resistance was associated with the upregulation of the RAS/MAPK pathway [133]. This suggested that the MAPK pathway may be a target for re-sensitization to PARPi. MEK inhibition (MEKi) was found to decrease cellular capacity for HR by decreasing the expression of MRE11, RAD50, NBN, and BRCA1/2 [133,134], predisposing to effects of PARPi. Combining MEKi and PARPi induced greater DNA damage and apoptosis in vitro, with synergistic anti-tumor activity in vivo [133,134]. An ongoing phase I/II trial of olaparib and selumetinib (MEKi) (NCT03162627) includes an expansion cohort of PARPi-resistant EOC.

### 8.5. PARPi and Inhibition of ATR, Chk1, and Wee1

Inhibitors of the ATR/Chk1/Wee1 axis affect both HR and replication fork stability, promoting re-sensitization to PARPi in settings of both BRCA1 and BRCA2 deficiency. In BRCA1-deficient cells, ATR inhibitors (ATRi) disrupted the acquired ATR-dependent recruitment of PALB2-BRCA2 and RAD51 loading [135,136], thereby reestablishing HR deficiency and overcoming the RAD51-related protection of stalled forks. The synergistic anti-tumor effect of combination PARPi and ATRi was demonstrated in PARPi-resistant BRCA1-mutant EOC models [137] and breast cancer models [138]. Furthermore, the role of ATR in coupling DNA damage repair with cell cycle regulation enhances the synergism of combination ATRi and PARPi, particularly in p53-mutated EOC [139]. In BRCA2-mutant EOC PDX models, ATRi released G2/M arrest, leading to premature mitosis with unrepaired PARPi-induced DNA damage [140]. This corresponded with increased tumor suppression in murine models. 

The inhibition of Chk1 (Chk1i), a downstream effector protein activated by ATR, produced similar results when administered with PARPi, in both BRCA mutant and wild-type EOC models [137,140,141]. Prexasertib, a Chk1 inhibitor, has demonstrated preclinical sensitization to PARPi [141,142] and early evidence of clinical effectiveness in a phase I trial [143]. Interestingly, two patients with PARPi-resistant, BRCA1-mutant HGSOC achieved partial responses with combination olaparib and prexasertib [143]. Effects of Chk1i may result from its known role interacting with Cdc25a and Cdc25c, possibly from effects on the transcription factor E2F7 [99], and by preventing RAD51 foci formation [141].

Acting downstream of Chk1, Wee1 inhibition synergizes with PARPi in preclinical data across several cancer types [144,145,146]. The safety of combination olaparib and the Wee1 inhibitor adavosertib was demonstrated in a phase 1b trial of refractory solid tumors [147], and this regimen is under investigation in a phase II trial specifically in PARPi-resistant EOC (NCT03579316). Interestingly, the sequential administration of PARPi and adavosertib was as effective as a concurrent administration in anti-tumor efficacy in vivo, but was better tolerated with fewer hematopoietic effects [146]. This alternate dosing schedule could be considered in future trials.

### 8.6. PARPi and BET/BRD4 Inhibition

Bromodomain containing 4 (BRD4) is a member of the BET protein family with roles in epigenetic gene regulation. The re-sensitization of PARPi-resistant cells using BRD4 inhibition (BRD4i) or broader BET inhibition (BETi) appears to be through the repression of HR-associated genes, including BRCA1, RAD51, and CtIP, thereby generating a state of HR deficiency [148,149,150]. Combined PARPi/BRD4i demonstrated antitumor synergy in vitro and in vivo [148,149,150]. This combination was effective in cell lineages that were BRCAwt (mimicking BRCA reversion mutations), 53BP1 deficient (mimicking TP53BP1 mutations and/or 53BP1 loss), and PARP1 deficient (mimicking PARP1 mutations or post-translational modifications), suggesting effectiveness across several mechanisms of PARPi resistance [148]. A phase I trial evaluating the combination of olaparib and AZD5153 (BETi) (NCT03205176) in advanced solid tumors, including EOC, allows prior PARPi exposure.

### 8.7. PARPi and CDK12 Inhibition

Cyclin-dependent kinase 12 (CDK12) modulates transcription by acting on RNA polymerase II. In vitro, CDK12 loss-of-function mutations and CDK12 inhibition (CDK12i) reduced the expression of HR-related genes, including BRCA1, ATR, and Fanconi-anemia pathway genes, due to premature cleavage and polyadenylation [151,152,153], leading to reduced capacity for HR repair [154,155,156]. This mechanism of re-inducing HR underlies the synthetic lethality between CDK12i and PARPi, seen in models of HGSOC [157] and breast cancer [156] and may be effective for PARPi-resistant disease in which HR is restored. There are thus far no clinical data for this combination.

### 8.8. PARPi and Immune Checkpoint Inhibition

Pairing PARPi and immune checkpoint inhibition (ICI) exploits two main premises for synergy. The first centers on the ability of PARPi to propagate DNA damage and generate cytosolic DNA. This activates the cGAS/STING pathway [158,159,160,161,162,163], with the downstream expression of type 1 interferons, T-cell-recruiting cytokines, and paracrine stimulation of dendritic cells [158,164]. However, this may be compromised if the mechanism of PARPi resistance is the restoration of HR proficiency. For example, downstream markers of cGAS/STING activation were decreased in BRCA proficient cells compared to BRCA deficient cells [158,165]. The second premise arises from the multiple immunomodulatory effects of PARPi, including on T cell differentiation, macrophage polarization [166,167,168], increased susceptibility to NK cell-mediated death [169,170], and PD-L1 upregulation [171,172]. These effects may be compromised if acquired alterations affect PARPi binding or allow the persistence of PARP1 function. The combination PARPi/ICI seeks to capitalize on the immunostimulatory effects of PARPi while negating the effects of PD-L1 upregulation.

The combination of PARPi/ICI may still be effective in the setting of restored HR proficiency. In the phase I/II TOPACIO/KEYNOTE-162 trial, the combination of niraparib and pembrolizumab was evaluated in patients with platinum-resistant EOC, of whom the majority were BRCAwt (79%) or HRP (53%) [173]. Overall response rates (ORR) were similar regardless of HR status; an ORR of 19% was seen in those patients with HRP disease, compared to an ORR of 14% in patients with HR deficient disease. This was comparable to the ORR of 14% (5/35) seen in a phase II trial of olaparib and durvalumab in a predominantly platinum-resistant (86%), BRCAwt (77%) patient population [174]. Of the five responders, two were BRCAwt and HRP. Paired pre- and on-treatment specimens showed that olaparib/durvalumab promoted an immunologically-inflamed environment, with increased IFNγ and TNFα production, increased tumor-infiltrating lymphocytes, and increased PD-L1 expression [174]. Therefore, though the clinical effectiveness of PARPi/ICI is modest in patients with measured or surrogate markers of HR proficiency, this combination may still be beneficial in select patients.

## 9. Conclusions and Future Perspectives

There are multiple mechanisms for PARPi resistance, and though many described mechanisms require clinical validation, the above data highlight several key issues. First, multiple resistance mechanisms may arise within one individual. This is well illustrated in the case of BRCA reversion mutations, in which several studies found numerous reversion mutations within individuals. The choice of subsequent PARPi combination therapy would need to consider this heterogeneity. Second, it is imperative to reassess the molecular characteristics of disease at each point of progression, given the dynamic nature of treatment response and resistance. “Liquid biopsy”, evaluating cell-free DNA (cfDNA), circulating tumors cells, circulating miRNA, or exosomes, may be more feasible than repeated biopsies [175]. For example, BRCA reversion mutations are readily detected using cfDNA [49,50,59,62]. Importantly, genetic alterations may have unanticipated consequences on the overall restoration of DNA repair or synthesis. In this regard, functional assays, such as those for the detection of HR deficiency/proficiency or replication fork stability, may be more informative than identifying the genetic changes in isolation. This is an area of unmet need, as surrogate measures of HR and replication fork dynamics, such as detection of γH2AX and Rad51 foci, are not yet clinically validated. Finally, PARPi combination therapies have great promise, but few trials currently allow patients with PARPi resistance. This is detrimental to the development of effective treatment strategies, particularly as the number of PARPi-resistant patients will rise with the growing use of PARPi in general. Ideally, post-progression PARPi combination regimens should complement the resistance mechanism(s) and functional status of the tumor at the time of treatment. To do so will require establishing the predictive value of both to optimize the pairing of patient and regimen. Expanding the benefit of PARPi through combination therapies will critically rely on appropriate dose escalation or alternative dose-schedule strategies. Optimizing target modulation while minimizing possible overlapping toxicities will prevent the premature termination of a promising combination. In conclusion, understanding the mechanisms of PARPi resistance, detecting them in real-time, such as through regular sampling by liquid biopsy, and optimizing targeted combinations, are critically needed.

## Abbreviations

altNHEJ	Alternative non-homologous end-joining
BER	Base excision repair
cfDNA	Cell-free DNA
cNHEJ	Classical non-homologous end-joining
DSB	Double strand break
EOC	Epithelial ovarian cancer
HGSOC	High grade serous ovarian cancer
HR	Homologous recombination
HRD	Homologous recombination deficient
HRP	Homologous recombination proficient
ICI	Immune checkpoint inhibition
NER	Nucleotide excision repair
NHEJ	Non-homologous end-joining
PARP	Poly(ADP-ribose) polymerase
PARPi	PARP inhibitor
PDX	Patient-derived xenograft
SSA	Single strand annealing
SSB	Single strand break
ssDNA	Single stranded DNA
VEGF/R	Vascular endothelial growth factor/receptor
